# Factors associated with participation in cardiac rehabilitation in patients with acute myocardial infarction: A systematic review and meta‐analysis

**DOI:** 10.1002/clc.24130

**Published:** 2023-08-18

**Authors:** Lingyu Wang, Jingyu Liu, Haiyan Fang, Xiang Wang

**Affiliations:** ^1^ Nursing School Anhui University of Chinese Medicine Hefei Anhui China

**Keywords:** acute myocardial infarction, cardiac rehabilitation, meta‐analysis, participation, related factors

## Abstract

**Background:**

Cardiac rehabilitation (CR) is effective in reducing morbidity and mortality in patients with acute myocardial infarction (AMI), but the participation rate is low and its influencing factors vary. Our study aimed to systematically review the literature and investigate the participation rates and influencing factors of CR in patients with AMI.

**Methods:**

We searched 10 databases, including PubMed, Web of Science, Cochrane Library, and so forth. A systematic review and meta‐analysis were conducted on the studies on the factors affecting CR participation in AMI. The *Q* tests and the *I* ^2^ tests were used to assess heterogeneity between studies. The combined effect size and odds ratio (OR) and their respective 95% confidence interval (CI) for CR participation rate and its influences are expressed, respectively. Stata 17.0 software was used for statistical analysis.

**Results:**

We included 14 studies with 114 542 participants. Current evidence indicates a CR participation rate of 34% (95% CI: 21%–46%) in patients with AMI. The pooled OR values and CI of each influencing factor are as follows: over 60 years old (OR = 0.865; 95% CI: 0.772–0.969), male (OR = 1.690; 95% CI: 1.276–2.239), college education or above (OR = 2.526; 95% CI: 1.117–5.711), ST‐segment elevation myocardial infarction (OR = 4.257; 95% CI: 2.004–9.045), decrease in left ventricular ejection fraction (OR = 0.918; 95% CI: 0.868–0.971), higher economic level (OR = 1.282; 95% CI: 1.108–1.483), history of coronary heart disease(OR = 0.667; 95% CI: 0.509–0.875), smoking (OR = 0.665; 95% CI: 0.550–0.805), combined hypertension (OR = 0.638; 95% CI: 0.562–0.723), and combined hyperlipidemia (OR = 0.577; 95% CI: 0.512–0.651).

**Conclusions:**

The overall participation rate of CR in AMI patients is low, and various factors affect the participation rate. Specialist medical staff are needed to further promote CR rehabilitation concepts and scientific knowledge, and take appropriate measures to address the influencing factors to increase CR utilization and improve patient prognosis.

## Graphical abstract

To investigate the current status and influencing factors of cardiac rehabilitation participation in patients with acute myocardial infarction.

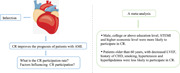

**Abbreviations**
AMI: acute myocardial infarction
CHD: coronary heart disease
CI: confidence interval
CR: cardiac rehabilitation
CVD: cardiovascular disease
ES: effect size
LVEF: left ventricular ejection fraction
OR: odds ratio
STEMI: ST‐segment elevation myocardial infarction

## 1 INTRODUCTION

Acute myocardial infarction (AMI) is characterized by acute onset, rapid progression, multiple complications and high mortality.^1^ Worldwide, more than 7 million people experience AMI each year, with a 1‐year mortality rate of approximately 10% and a second cardiovascular event in the first year in 20% of those who survive.^2^ These figures suggest that we need to take appropriate measures and that prevention after AMI is essential to reduce risk and suffering. Studies have shown that cardiac rehabilitation (CR) can reduce morbidity and mortality and improve the quality of life of people with heart disease.^3^, ^4^

CR is an evidence‐based intervention that uses patient education, health behavior modification, and exercise training to improve secondary prevention outcomes in patients with cardiovascular disease (CVD).^4^ It is recommended by international guidelines as Level 1A evidence for use in cardiovascular patients.^5^ However, studies have shown that CR is not fully utilized and its participation rate is low^6^, ^7^ although these studies have outlined the reasons for this, the results are mixed and our understanding of the causes remain incomplete. This meta‐analysis aimed to understand the current status of CR participation rates in AMI patients and the factors that influence them. The study has important implications for increasing CR utilization, improving the prognosis of AMI patients, and improving quality of life.

## 2 METHODS

### 2.1 Protocol and registration

We conducted this study under the Preferred Reporting Items for Systematic Reviews and Meta‐Analyses,^8^ registration number: CRD42023367431.

### 2.2 Search strategy and selection criteria

We searched PubMed, Web of Science, Cochrane Library, Medline, Embase, Scopus, China National Knowledge Infrastructure (CNKI), China Science and Technology Journal Database (VIP), China Biomedical Literature Database (CBM), and Wanfang Database by computer. Articles on CR participation rates and their influencing factors in AMI patients were collected, and the search time frame was from the creation of the database to October 2022. The search terms were “AMI,” “MI,” “CR,” “participation,” “related factors,” “influencing factors,” “factors,” and “reasons.” In addition, we searched the reference lists of included studies as a way to identify other articles that might be included. Our criteria for selecting articles were (a) participants were patients (age >18 years) diagnosed with AMI, including those recovering from hemodynamic reconstruction; (b) the study is examining the rate of CR participation in AMI patients and the factors that influence it. CR involvement is defined as CR registration and treatment during hospitalization or after discharge, and can be any stage of CR; and (c) the study design was a cross‐sectional study, cohort study, or case‐control study. Our criteria for excluding articles were (a) duplicate publications (only the one with the most information retained); (b) studies with incomplete data or unavailable full text; and (c) studies such as conferences, reviews, animal studies, and unpublished studies. The selection of articles was done by two authors, and in case of disagreement, three authors discussed and negotiated the final results.

### 2.3 Comorbidity variables

We chose the definition of comorbidity variables to be consistent in the process of inclusion of studies. (a) Combined hyperlipidemia: refers to patients with clinically diagnosed AMI who have hyperlipidemia and only these two diseases; (b) combined hypertension: refers to patients with clinically diagnosed AMI who have hypertension and only these two diseases; (c) history of coronary heart disease (CHD): a history of previous CHD in the patient.

### 2.4 Data extraction and quality assessment

Data extraction and quality assessment of articles were carried out by two researchers relatively independently and cross‐checked. In case of disagreement, it was discussed and negotiated through three researchers. The results were extracted uniformly as follows the name of the first author, year of publication, country, study design, sample size, influencing factors and their odds ratios (ORs), 95% confidence interval (CI), and so forth. The Newcastle–Ottawa Scale (NOS)^9^ was used to evaluate the quality of the included cohort. The total score of the scale was 0–9, and the total score ≥7 was considered to be of high quality. The included cross‐sectional studies were evaluated using the Agency for Healthcare Research and Quality (AHRQ)^10^ evaluation criteria. The total score of AHRQ was 0–11, and the total score was 0–3 for low quality, 4–7 for moderate quality, and 8–11 for high quality.

### 2.5 Statistical analysis

We used Stata 17.0 software to complete the corresponding statistical analyses. In this study, the combined effect value of CR participation rate was expressed as effect size (ES) (95% CI), and the combined effect value of its influencing factors was expressed as OR (95% CI); if *p* < .05 indicated that the combined ES were statistically significant. Heterogeneity between studies was assessed by *I* ^2^ and *Q* tests; if *I* ^2^ < 50% and *p* > .05, homogeneity between multiple studies was considered to exist and a fixed‐effects model was used; conversely, a random‐effects model was used. Sensitivity analysis was used to determine the source of heterogeneity, and the consistency of the results between the fixed effect model and the random effect model was compared. If the results were inconsistent, the articles that had a greater impact on the results needed to be excluded for further analysis. Egger's test was used to judge publication bias, if *p* > .05, indicating no publication bias.

## 3 RESULTS

### 3.1 General study characteristics

**Figure 1:**
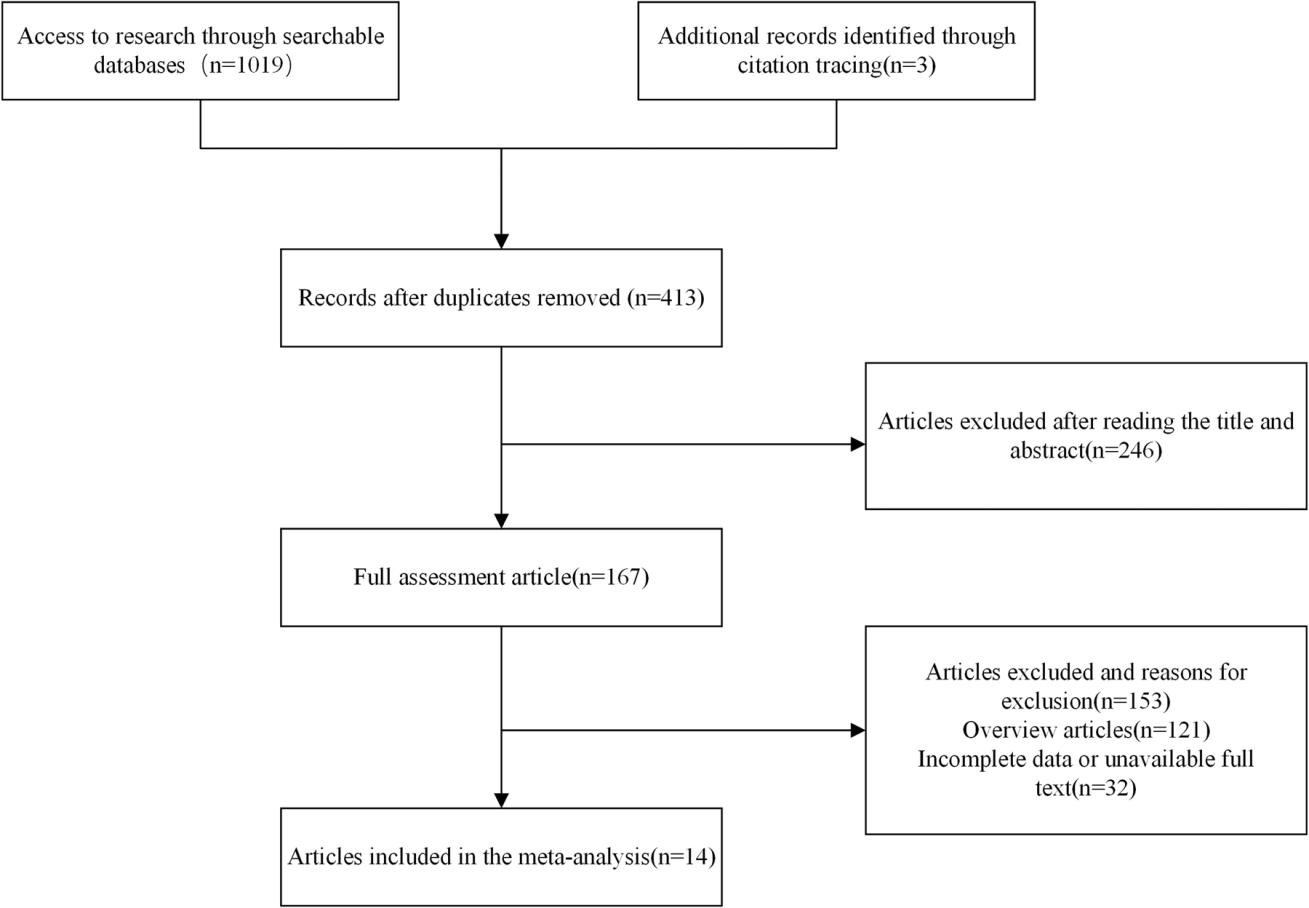
Flow chart of literature screening process and results.

A total of 1022 articles were collected from the database, of which three articles were obtained by tracking citations. Duplicates were removed to obtain 413 articles. A total of 246 articles were excluded after reading the titles and abstracts, then the full texts of 167 articles were read, 153 articles were excluded, including 121 review articles and 32 articles with incomplete data, and 14^11^, ^12^, ^13^, ^14^, ^15^, ^16^, ^17^, ^18^, ^19^, ^20^, ^21^, ^22^, ^23^, ^24^ articles were finally included. The detailed process is shown in Figure 1. Eleven of the included studies were cohort studies and three articles were cross‐sectional studies, both of medium to high quality. A total of 114 542 participants were included in the studies. All included studies were published between 2017 and 2022 and were conducted in six countries (China, Netherlands, Korea, Switzerland, Denmark, and the United States). The basic characteristics and quality assessment of the included studies are shown in Table 1.

**Table 1:** Basic information about the included studies.

Author	Year	country	Study design	Sample size (cases)	Number of participants (examples)	Participation rate (%)	Quality score
Wu et al.^11^	2019	China	Cohort studies	483	63	13.04	7
Gui et al.^12^	2022	China	Cohort studies	1365	409	29.96	7
Xu et al.^13^	2022	China	Cohort studies	454	32	7.05	7
Zhang et al.^14^	2021	China	Cohort studies	252	65	25.79	7
Ma et al.^15^	2021	China	Cohort studies	472	104	22.03	8
Sunamur et al.^16^	2017	The Netherlands	Cohort studies	3871	1497	39	6
Gonzalez‐Jaramillo et al.^17^	2022	Switzerland	Cohort studies	3011	1058	35	7
Kim et al.^18^	2020	Korea	Cohort studies	64 982	960	1.50	7
Brouwers et al.^19^	2020	The Netherlands	Cohort studies	640	371	58	8
Peter and Keeley^20^	2020	The Netherlands	Cross‐sectional	32 792	10 494	32	8
Graversen et al.^21^	2017	Denmark	Cross‐sectional	3356	2062	61.40	9
Rodrigo et al.^22^	2021	The Netherlands	Cohort studies	469	353	75	6
Pedersen et al.^23^	2018	Denmark	Cohort studies	302	91	30	7
Kianoush et al.^24^	2022	United States	Cross‐sectional	2093	887	42.4	8

### 3.2 Results of the CR participation rate meta‐analysis

**Figure 2:**
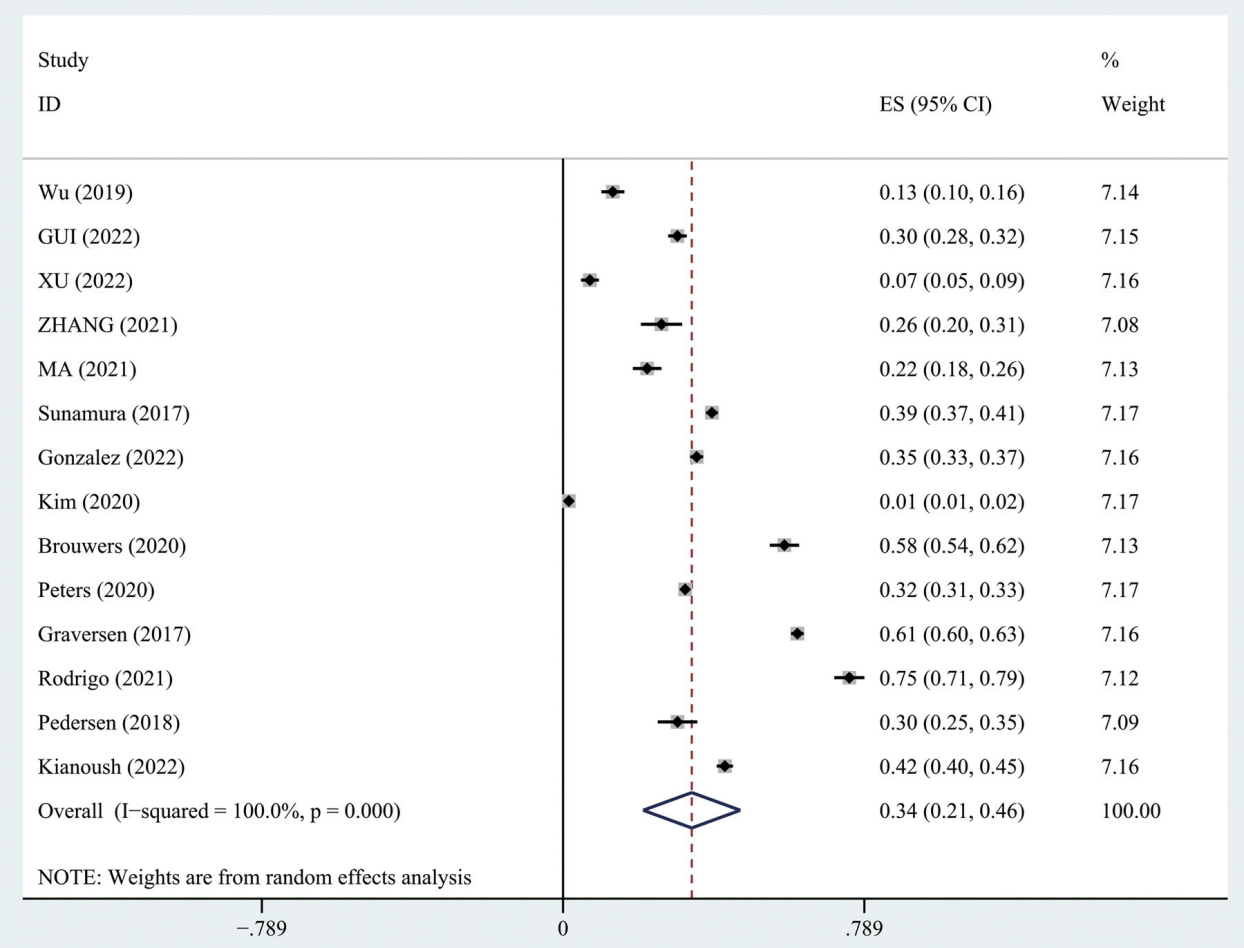
Cardiac rehabilitation participation rate in patients with acute myocardial infarction.

Meta‐analysis showed a CR participation rate of 34% (95% CI: 21%–46%) in patients with AMI (Figure 2).

### 3.3 Results of a meta‐analysis of factors associated with factors affecting participation in CR

In this study, we only extracted the factors involved in two or more articles for meta‐analysis, so 12 influencing factors were finally extracted. The results showed that AMI patients who were male, education level of university or above, ST‐segment elevation myocardial infarction (STEMI), and higher economic level were more likely to participate in CR, while AMI patients over 60 years of age, a decrease in left ventricular ejection fraction (LVEF), a history of CHD, smoking, combined hypertension, and combined hyperlipidemia were less likely to participate in CR. Distance from the CR center and insurance was not associated with CR participation in AMI patients (Table 2).

**Table 2:** Meta‐analysis of factors influencing participation in CR.

Influencing factors	Number of studies	Effect size		Test of heterogeneity		Model	Bias of publication
		OR (95% CI)	*p*‐Value	*I* ^2^ (%)	*p*‐Value		Egger's test (*P*)
Over 60 years old	10	0.865 (0.772, 0.969)	.012	96.5	<.001	Random effects model	0.349
Male	5	1.690 (1.276, 2.239)	<.001	79.7	.001	Random effects model	0.404
College education or above	6	2.526 (1.117, 5.711)	.026	96.2	<.001	Random effects model	0.183
STEMI	5	4.257 (2.004, 9.045)	<.001	96.5	<.001	Random effects model	0.326
Decrease in LVEF	2	0.918 (0.868, 0.971)	.003	61.8	.106	Fixed effects model	—
Higher economic level	2	1.282 (1.108, 1.483)	.001	0.0	.800	Fixed effects model	—
History of CHD	2	0.667 (0.509, 0.875)	.003	62.3	.103	Fixed effects model	—
Smoking	2	0.665 (0.550, 0.805)	<.001	39.7	.198	Fixed effects model	—
Distance from the CR center	3	1.076 (0.537, 2.157)	.836	80.4	.006	Random effects model	0.979
Combined hypertension	2	0.638 (0.562, 0.723)	<.001	12.1	.286	Fixed effects model	—
Combined hyperlipidemia	3	0.577 (0.512, 0.651)	<.001	0.0	.480	Fixed effects model	0.093
Insurance	2	2.614 (0.108, 63.313)	.555	97.7	.000	Random effects model	—

Abbreviations: CHD, coronary heart disease; CI, confidence interval; CR, cardiac rehabilitation; LVEF, left ventricular ejection fraction; OR, odds ratio, STEMI, ST‐segment elevation myocardial infarction.

### 3.4 Publication bias test

The Egger test was performed on the factors affecting participation in CR to judge the publication bias of the included studies. The test results showed that *p* > .05 for each influencing factor, indicating that there was little possibility of publication bias among the studies. The specific results are shown in Table 2.

### 3.5 Sensitivity analysis

Twelve factors affecting CR participation were analyzed by the random effect model and fixed effect model, respectively. The results showed that except for age, education level of college or above, decreased LVEF function, history of CHD, and distance to CR center, the combined effect values of other factors were not significantly different, indicating that the results of Meta‐analysis were relatively stable (Table 3).

**Table 3:** Sensitivity analysis of factors influencing the participation of patients with AMI in cardiac rehabilitation.

Influencing factors	Random effects model	Fixed effects model
Over 60 years old	0.865 (0.772, 0.969)	0.989 (0.975, 1.003)
Male	1.690 (1.276, 2.239)	1.792 (1.609, 1.997)
College education or above	2.526 (1.117, 5.711)	1.012 (0.896, 1.142)
STEMI	4.257 (2.004, 9.045)	2.535 (2.258, 2.847)
Decrease in LVEF	0.843 (0.647, 1.099)	0.918 (0.868, 0.971)
Higher economic level	1.282 (1.108, 1.483)	1.282 (1.108, 1.483)
History of CHD	0.587 (0.329, 1.047)	0.667 (0.509, 0.875)
Smoking	0.667 (0.522, 0.852)	0.665 (0.550, 0.805)
Distance from the CR center	1.076 (0.537, 2.157)	1.030 (1.015, 1.045)
Combined hypertension	0.629 (0.531, 0.746)	0.638 (0.562, 0.723)
Combined hyperlipidemia	0.577 (0.512, 0.651)	0.577 (0.512, 0.651)
Insurance	2.614 (0.108, 63.313)	0.819 (0.590, 1.135)

Abbreviations: AMI, acute myocardial infarction; CHD, coronary heart disease; CR, cardiac rehabilitation; LVEF, left ventricular ejection fraction; STEMI, ST‐segment elevation myocardial infarction.

## 4 DISCUSSION

The 14 studies involved six countries and the meta‐analysis showed that the CR participation rate for AMI patients was 34% (95% CI: 21%–46%). The economic level, cultural background, and medical conditions vary between countries, which may affect the CR participation rate of AMI patients.

This study showed that age, education level of university or above, and higher economic level were the influencing factors of CR participation in AMI patients. Older people (over 60 years) were less likely to participate in CR, while patients with college or above educational level and high economic level were more likely to participate in the CR program. Related studies have shown that the elderly have a relatively low educational level (primary level and below) and a decline in cognitive ability, which affects their cognitive level during CR programs. Secondly, the lack of financial independence (retirement) of older people and the lack of accessibility are also constraints, which explain the low participation rate of older patients in CR.^25^ The commuting time and distance to the CR center were the barriers to CR treatment, but different geographical features and areas with easy access to public transport vary.^23^ However, the influencing factor of distance was not significant in this study. Moradi et al.^26^ suggested that patients with a low level of education could not fully understand the content of CR health education and the behavior change requested of them by the medical staff. In addition, patients with a low level of education are prone to insecurity and need assistance from others for CR, which is not conducive to their long‐term adherence.^27^ Graversen et al.^21^ showed that a low level of socioeconomic status, receiving less relevant information about CR, and a lower intention to participate in CR. Data showed that patient CR participation rates were associated with microeconomic income, with patients with low income as well as those without insurance have lower participation rates than those with high income and insurance.^28^ In this study, insurance, an influencing factor, did not have a significant effect on CR participation rates. It is suggested that in the future, while improving CR utilization, researchers should further explore personalized CR for patients of different age groups, literacy levels, and economic levels, and healthcare workers need to personalize CR content to suit the characteristics of different patient groups to ensure that AMI patients benefit from CR.

This study suggests that men are more likely to participate in CR than women. Although CR has proven benefits in reducing CVD‐related mortality, improving quality of life, and preventing readmissions, women who complete CR programs achieve similar physical benefits as men and may experience greater improvements in psychosocial well‐being, CR is underutilized relative to men.^29^, ^30^ Li et al.^31^ used the American Heart Association's Coronary Artery Disease registry and found that women were 12% less likely to be referred to CR than men. Ritchey et al.^32^ found that women were 9% and 13% more likely to attend and complete CR, respectively. At present, the majority of CR targets are not gender‐specific, and the differences in clinical characteristics, physical characteristics, psychosocial characteristics, and family responsibilities caused by gender differences are not considered, which may be the reason for the lower CR participation rate of women than men. We look forward to further research on CR in the future, and gradually establishing a female‐centered CR based on gender differences, to improve the participation rate and utilization rate of CR in women and improve the prognosis of female AMI patients.

Disease type and comorbidities were also factors affecting the CR participation rate. Previous studies have reported that patients with multiple comorbidities will greatly reduce their probability of participating in CR,^33^ and have a negative impact on compliance with CR plans. Dunlay et al.^34^ noted that participants with CR had a lower prevalence of hypertension than nonparticipants and a greater probability of participating in CR with STEMI. This is consistent with the results of the present study. Guidelines recommend CR as a treatment for patients with coronary artery disease, regardless of left ventricular systolic function. However, patients with reduced LVEF were less likely to participate in CR, which may be related to patients' fear of worsening their condition.^35^ However, some studies have found that patients with reduced LVEF have a greater benefit from participating in CR compared with patients with normal LVEF.^36^ Kotseva et al.^37^ showed that patients with pre‐existing CHD and hypertension were less likely to participate in CR. It may be related to the patient's fear of reoccurrence of adverse cardiovascular events in the process of participating in CR, potential threat to life safety, and persistent fear of the future and death. Hyperlipidemia is one of the main risk factors for CHD. The effect of simple diet control on hyperlipidemia is not as good as exercise plus diet control. The results of a recent systematic review suggest that exercise programs in CR are beneficial in terms of lipid improvement, reducing lipid levels in plasma.^38^ A meta‐analysis has also shown that exercise‐based CR reduces lipid levels.^39^ Based on the above evidence, CR has a positive effect on patients with hyperlipidemia, but the results of this study suggest that patients with hyperlipidemia have a low probability of participating in CR. The reasons for this are poorly understood and need further research.

Smoking is the most important and modifiable risk factor for CVD, and continued smoking after a major cardiac event predicts worse health outcomes and leads to reduced participation in CR.^40^, ^41^ Studies have shown that smokers are consistently less likely to participate in or complete CR than nonsmokers.^42^ This is consistent with the results of this study. Although smokers are referred to CR more frequently, they have a lower probability of participating in CR programs and a higher dropout rate.^43^ Smoking is not only a risk factor for CHD, but also can significantly weaken the confidence of patients to adhere to CR. Previous studies have shown a significant correlation between smoking and withdrawal from rehabilitation exercises.^44^ In view of this phenomenon, medical staff should strengthen the popularization of the harm of smoking to their condition in hospitalized patients with AMI, and encourage patients to actively participate in CR. Researchers should consider the addiction of smoking patients and design future studies to adapt CR for smoking patients.

Our study has the following strengths. First, we conducted a comprehensive database search and traced relevant citations, which ensured that the literature was comprehensively searched. Second, in the process of data extraction and data processing, two researchers independently processed and cross‐checked, and the third researcher was consulted in time when there was any disagreement, to ensure the accuracy of the data and strictly control the quality. Finally, we conducted a publication bias test and sensitivity analysis on the included studies. However, our study has several limitations. First, although we comprehensively searched the database, we still missed some grey kinds of literature. Second, we only analyzed the influencing factors of the CR participation rate in six countries, and the results may not be representative of the CR participation rate in all countries. Third, the included studies had some variation in sample size, which may have caused bias; therefore, the results should be interpreted with caution.

## 5 CONCLUSIONS

The results of this meta‐analysis showed that the overall CR participation rate of AMI patients was low, and it is necessary for specialist medical staff to further promote the concept of CR and popular science knowledge to improve the utilization rate of CR. In addition, AMI patients who were male, education level of university or above, STEMI, and higher economic level were more likely to participate in CR, while AMI patients over 60 years of age, with a decrease in LVEF, a history of CHD, smoking, combined hypertension, and combined hyperlipidemia were less likely to participate in CR. Distance from the CR center and insurance was not associated with CR participation. Due to the quality, quantity, and regional limitations of the included studies, large sample, multiregional and multicenter studies are needed to explore the factors associated with CR participation rates in AMI patients.

## AUTHOR CONTRIBUTIONS

Lingyu Wang and Jingyu Liu conceived the study, designed the study, and wrote the paper. Lingyu Wang, Jingyu Liu, and Haiyan Fang extracted the data. Lingyu Wang, Jingyu Liu, and Xiang Wang analyzed the data. Haiyan Fang made significant revisions to the manuscript and all authors approved the final manuscript.

## CONFLICT OF INTEREST STATEMENT

The authors declare no conflict of interest.

## Data Availability

All data generated or analyzed in this study are included in the text.
